# Psychological distress in young adults with well-controlled psoriasis

**DOI:** 10.3389/fpsyt.2025.1753103

**Published:** 2026-01-12

**Authors:** Eva Klara Merzel Šabović, Tadeja Kraner Šumenjak, Miodrag Janić

**Affiliations:** 1 Department of Dermatovenerology, University Medical Centre Ljubljana, Ljubljana, Slovenia; 2 Faculty of Medicine, University of Ljubljana, Ljubljana, Slovenia; 3 Faculty of Agriculture and Life Sciences, University of Maribor, Hoče, Slovenia; 4 Clinical Department of Endocrinology, Diabetes and Metabolic Diseases, University Medical Centre Ljubljana, Ljubljana, Slovenia

**Keywords:** psoriasis, HADS, depression, anxiety, treatment, young patients

## Abstract

**Introduction:**

Psychological distress is common in psoriasis, but its prevalence and possible biological correlates in young adults with well-controlled disease remain unclear. In this study, we aimed to assess the presence of anxiety and depressive symptoms in young, well-treated psoriasis patients, who were primarily expected to have no or minimal psychological distress, and to explore potential associations with inflammatory and metabolic markers.

**Methods:**

In a cross-sectional cohort of 80 psoriasis patients (women/male: 45/35; mean age 38.7 ± 4.2 years) psychological symptoms were assessed using the Hospital Anxiety and Depression Scale (HADS), with HADS-A and HADS-D subscales evaluating anxiety and depression symptoms. Exploratory associations with inflammatory cytokines and metabolic indices were examined using regression analyses and random forest machine-learning models.

**Results:**

Anxiety symptoms were identified only in 5 patients (women/male: 4/1; 6%), and depressive symptoms in 10 patients (women/male: 6/4; 12%). Affected individuals had clinically significant anxiety (HADS-A: 12/21) and borderline depressive symptoms (HADS-D: 10/21). No significant associations between HADS scores and inflammatory or metabolic markers were found. Exploratory random forest models tentatively identified IL-6, IL-23, FIB-4, HOMA-IR, and waist circumference as the strongest contributors to variance in depressive symptoms, while no clear contributors emerged for anxiety.

**Discussion:**

Anxiety and depressive symptoms can occur in well-treated young psoriasis patients. Although prevalence is low, their impact may be substantial. These findings indicate the potential importance of targeted psychological screening in this population. Associations with inflammatory and metabolic markers should be considered hypothesis-generating and warrant validation in larger, independent cohorts.

## 1 Introduction

Psoriasis is a chronic immune-mediated disease that can substantially affect mental health (1–3). Reported prevalence rates of depression in people with psoriasis range from 8.5% to 23% (4–6), while anxiety affects approximately 16.9% to 36% of patients (1, 7, 8). The development of depression and anxiety in psoriasis is complex and multifactorial, likely influenced by disease severity, lesion location, genetic susceptibility, neuroendocrine imbalances, psychological stressors, systemic inflammation, metabolic disorders, comorbidities, and altered gut microbiota (5, 6, 9). Since disease severity and visible skin lesions are recognized as major triggers of psychological distress, it is often assumed that patients with well-controlled disease and cleared skin experience minimal mental health burden. However, this assumption may overlook emotional challenges in this presumed low-risk group.

The Hospital Anxiety and Depression Scale (HADS) is a widely validated tool for assessing psychological distress in clinical populations, comprising separate subscales for anxiety (HADS-A) and depression (HADS-D), each ranging from 0 to 21 (10, 11). Scores of 8–10 indicate borderline symptoms, while scores ≥11 suggest clinically significant anxiety or depression (10). HADS has been applied in various patient groups, including those with psoriasis and psoriatic arthritis (11, 12). However, despite its validation and recommendations for routine use, HADS remains underutilized in dermatological practice.

In the present study, we aimed to investigate the presence of anxiety and depressive symptoms, as measured by HADS-A and HADS-D, in young adults with effectively treated psoriasis, a group generally considered “low-risk” but nonetheless psychologically vulnerable, for which few data are currently available. We also tentatively explored potential associations, or signals thereof, between HADS scores and inflammatory and metabolic markers, both closely linked to psoriasis-related comorbidities, using conventional regression analyses and random forest machine-learning models.

## 2 Materials and methods

### 2.1 Study population and design

We conducted a cross-sectional study involving 80 patients with psoriasis (54 men, 26 women) at the Dermatology Outpatient Clinic, Department of Dermatovenerology, University Medical Centre Ljubljana, Slovenia. Patients were consecutively recruited and had been effectively treated with topical therapy (n = 21) or systemic therapy, including methotrexate (n = 11), adalimumab (n = 14), secukinumab (n = 14), or guselkumab (n = 20). The efficacy of the treatment was evaluated using the Psoriasis Area and Severity Index (PASI). A total of 87.5% of the patients achieved excellent disease control (PASI < 3), with 46% achieving complete clearance (PASI = 0), and 12.5% showing a good response (PASI 5–7). All patients received standard clinical care, and none had been previously diagnosed with anxiety or depression. Routine psychological screening was not part of standard management. Inclusion and exclusion criteria were extensively defined in previous publications (13–16). Informed consent in writing was obtained from all participants. The study was approved by the Slovenian National Medical Ethics Committee (approval no. 0120-422/2021/6) and carried out in accordance with the Declaration of Helsinki (1975, revised 2013). It is registered on ClinicalTrials.gov (Identifier: NCT05957120) and follows the STROBE guidelines (17).

### 2.2 Study protocol including HADS assessment

During the study visit, a comprehensive medical history was obtained and a physical examination was conducted. Anthropometric measurements were recorded, and fasting blood samples were collected. Psychological status was assessed using the validated Hospital Anxiety and Depression Scale (HADS), a 14-item self-report questionnaire with two subscales: seven items measuring anxiety (HADS-A) and seven measuring depression (HADS-D). Each item is scored from 0 to 3, resulting in subscale scores ranging from 0 to 21. Scores of 0–7 are considered normal, 8–10 indicate borderline cases, and ≥11 reflect clinically significant anxiety or depression (10).

### 2.3 Laboratory methods and calculation of metabolic indices

Laboratory samples were processed according to standardized procedures and glycated hemoglobin (HbA1c), biochemical [fasting glucose, insulin, total cholesterol, LDL (low-density lipoprotein) cholesterol, HDL (high-density lipoprotein) cholesterol, triglycerides, alanine aminotransferase (ALT), and aspartate aminotransferase (AST)] and inflammatory [high-sensitivity C-reactive protein (hs-CRP), inflammatory cytokines: tumor necrosis factor (TNF), interferon-γ (IFN-γ), interleukin (IL)-1β, IL-6, IL-12p70, IL-17, and IL-23)] parameters were determined, as extensively described previously (13, 15). Metabolic indices including Homeostatic Model Assessment of Insulin Resistance (HOMA-IR), Triglyceride-Glucose Index (TyG), and Fibrosis-4 Index (FIB-4) were calculated (13).

### 2.4 Statistical analysis

Bivariate associations between HADS scores and covariates (BMI, waist circumference, HbA1c, HOMA-IR, FIB-4, TyG index, hs-CRP, TNF, IFN-γ, IL-1β, IL-6, IL-12p70, IL-17, IL-23) were assessed using Spearman’s rank correlation, with correlation coefficients, p-values, and 95% confidence intervals reported. P-values were Holm-adjusted for multiple comparisons. Multiple linear regression was performed with Box-Cox–transformed outcomes to meet normality assumptions, adjusting for age, gender, psoriasis duration, treatment duration, treatment type, and smoking status. Regression coefficients, confident intervals, and p-values were extracted.

A random forest model was used to explore metabolic, inflammatory, demographic, and clinical covariates in relation to HADS, HADS-A, and HADS-D scores. The model provides estimates of variable importance, allowing identification of the biomarkers or clinical indicators that contribute most strongly to these outcomes. Class imbalance was addressed with inverse-frequency weighting. Model performance was evaluated via permutation-based variable importance (Mean Decrease Accuracy) and multiclass ROC curves (AUC), with AUC <0.5 indicating no predictive signal. Variables causing greater accuracy loss were considered more important. All analyses were performed in R (v3.6.1).

## 3 Results

The characteristics of patients are presented in **Table 1**. Of the 80 patients included, 30 (37%) were smokers.

**Table 1:** Characteristics of patients.

Patients’ characteristics		TOP (n=21)	Systemic therapy			
			MTX (n=11)	ADA (n=14)	SEC (n=14)	GUS (n=20)
Age (years)		38.00 (32.00-41.50)	39.00 (35.00-42.00)	39.50 (36.75-41.00)	39.50 (34.50-43.25)	40.00 (36.00-43.00)
Sex	Male	13	7	10	11	13
	Female	8	4	4	3	7
Smokers No. (%)		7 (33)	3 (27)	7 (50)	8 (57)	4 (20)
Duration of psoriasis (years)		8.0 (4.5-20.0)	10.0 (5.0-12.0)	20.0 (11.5-25.0)	16.5 (13.75-23.25)	20.0 (15.0-23.5)
Duration of treatment (months)		77.0 (47.0-239.0)	29.0 (21.0-47.0)	95.0 (61.0-117.5)	48.0 (33.5-59.0)	31.0 (27.3-51.0)
BMI (kg/m^2^)		23.36 (22.59-26.18)	28.05 (22.65-37.51)	27.04 (23.62-30.66)	31.04 (26.75-35.39)	27.50 (24.54-34.96)
Waist circumference (cm)		86.00 (77.50-94.50)	104.00 (92.00-121.50)	93.25 (89.125-109.375)	104.50 (98.00-113.50)	99.25 (85.625-108.875)
PASI		0.2 (0.1-1.75)	1.2 (0.7-3.2)	0.0 (0.0-0.2)	0.0 (0.0-0.65)	0.0 (0.0-0.6)
HADS	normal	21	10	13	14	18
	bordeline	0	1	1	0	1
	clinical	0	0	0	0	1
HADS-D	normal	20	11	14	14	18
	bordeline	1	0	0	0	2
	clinical	0	0	0	0	0
HADS-A	normal	17	7	13	12	18
	bordeline	4	2	0	2	0
	clinical	0	2	1	0	2

TOP, topical therapy; MTX, methotrexate; ADA, adalimumab; SEC, secukinumab; GUS, guselkumab; HADS, Hospital Anxiety and Depression Scale; BMI, Body Mass Index; PASI, Psoriasis Area and Severity Index. *Data are presented as the median (interquartile range), or number of cases for categorical variables*.

The bivariate associations between HADS scores and hs-CRP, TNF, IFN-γ, IL-1β, IL-6, IL-12p70, IL-17, IL-23, BMI, waist circumference, HbA1c, HOMA-IR, FIB-4 and TyG index, were analyzed; however, none reached statistical significance. Similarly, in multiple linear regression models adjusted for age, sex, duration of psoriasis, duration of treatment, type of treatment (topical vs. systemic), and smoking status, no significant associations were observed.

**Figure 1:**
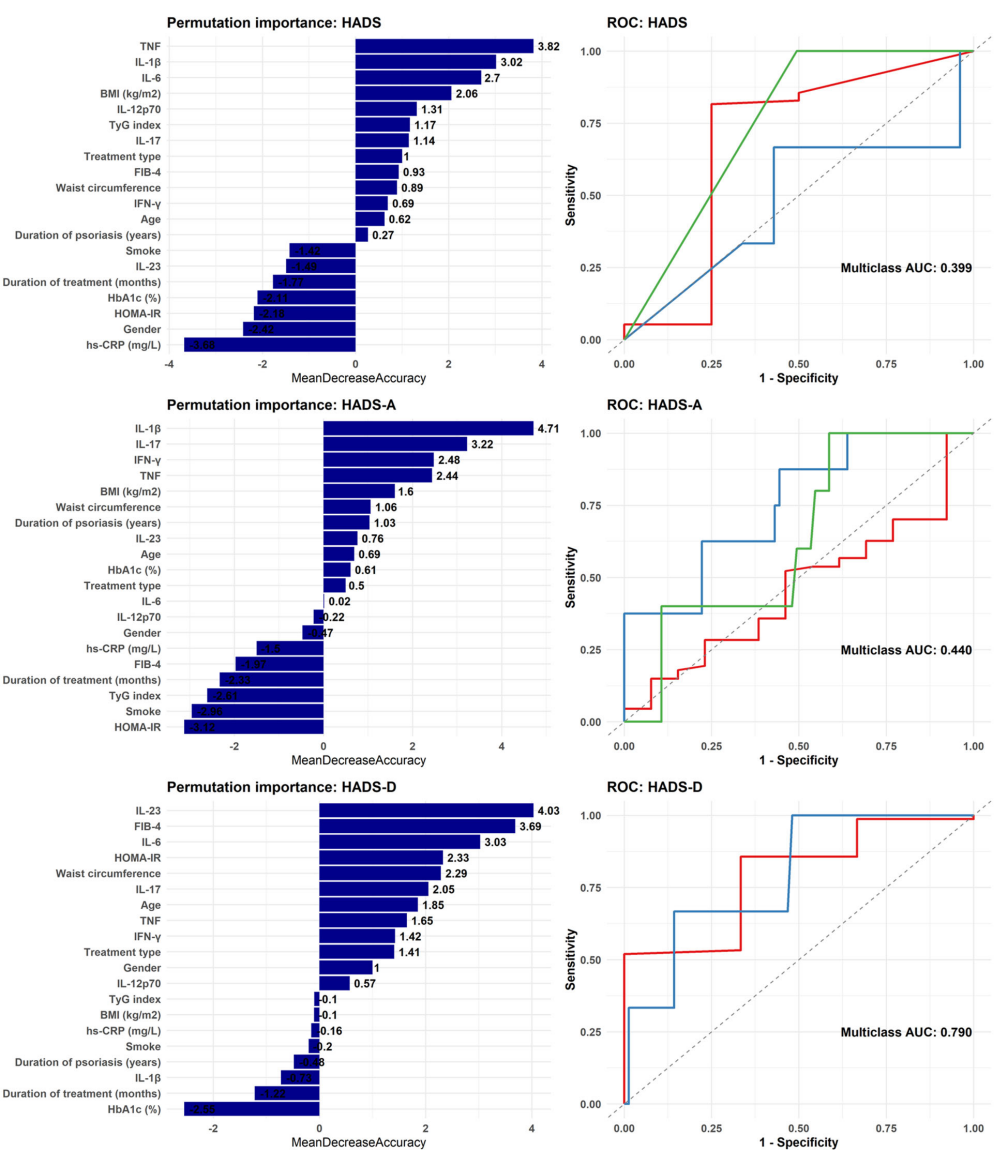
One-versus-all ROC curves and permutation importance plots for random forest models predicting HADS outcomes. ROC curves (right) and variable importance (left) are shown for models trained to classify total HADS score, HADS-Anxiety (HADS-A), and HADS-Depression (HADS-D). Each model was trained using a combined set of inflammatory markers (TNF, IFN-γ, IL-1β, IL-6, IL-17, IL-23, hs-CRP), metabolic markers (BMI, waist circumference, HbA1c, FIB-4, TyG index, HOMA-IR), and demographic/clinical variables (duration of psoriasis, age, sex, treatment type, duration of treatment, smoking status). Multiclass AUC values are reported for each ROC curve. *hs-CRP, high sensitivity C-reactive protein; TNF, tumor necrosis factor; IFN-γ, interferon-γ; IL, interleukin; BMI, body mass index; HbA1c, glycated hemoglobin; HOMA-IR, homeostatic model evaluation for insulin resistance; FIB-4, fibrosis-4 index for liver fibrosis; TyG index, triglyceride glucose index*.

Random forest models were then used to predict HADS, HADS-A, and HADS-D categories based on metabolic and inflammatory biomarkers, as well as demographic and clinical covariates (**Figure 1**). The HADS and HADS-A models demonstrated poor discriminative ability, with AUCs of 0.399 and 0.440, respectively, indicating performance no better than random guessing. This suggests that anxiety symptoms in this cohort may have a multifactorial basis not adequately captured by the selected biomarkers. In contrast, the HADS-D model showed substantially higher discrimination (AUC = 0.790). For this outcome, permutation-based variable importance (Mean Decrease Accuracy, MDA) identified IL-6, IL-23, waist circumference, HOMA-IR, and FIB-4, as the strongest predictors. Additional relevant variables included age, IL-17, TNF, and IFN-γ, while others such as BMI, hs-CRP, TyG index, duration of psoriasis, duration of treatment, HbA1c, smoking, and IL-1β demonstrated negligible or negative importance, indicating limited contribution to model performance. Given the small sample size and the absence of significant correlations, the results of the random forest analysis can be only considered hypothesis-generating rather than conclusive.

## 4 Discussion

In this study, we observed that a small subset of young adults with well-controlled psoriasis continued to experience psychological distress, even in the absence of the primary trigger—visible skin lesions. 5% had elevated overall HADS scores, 16.2% had elevated HADS-A scores, and 3.7% had elevated HADS-D scores, with a predominance of women across categories. Overall, roughly one in five patients in this presumably low-risk group experienced psychological distress. Notably, these anxiety and depressive symptoms were neither reported by the patients nor recognized by their dermatologists or primary care physicians. These findings highlight the importance of proactive psychological screening as an integral part of routine care for this patient group.

The novelty of our study lies in its focus on young adults with well-controlled psoriasis, a population generally expected to experience minimal psychological distress. Unlike most previous studies that include mixed-age or treatment-naïve cohorts, we specifically examined whether anxiety and depressive symptoms persist in younger patients (mean age 38.1 ± 4.0 years) even when their physical disease is effectively managed. Our cohort included patients with moderate to severe psoriasis, receiving treatments ranging from topical therapies to biologics (namely topical treatment, methotrexate, adalimumab, guselkumab and ustekinumab), thereby reflecting a representative spectrum of young adults with psoriasis in real-world clinical practice.

It is generally accepted that anxiety and depression are common among psoriasis patients, regardless of age or disease activity (18). A study of 44,838 adolescents reported that those with psoriasis had a significantly higher risk of depressive symptoms (odds ratio 1.38) compared with peers without the condition (19). Importantly, most published studies have examined anxiety and depression as separate outcomes. A systematic review of 56 studies found that the prevalence of depression and anxiety among adults with psoriasis was 20% and 21%, respectively, highlighting the substantial psychological burden of the disease across all age groups (20). Collectively, our results reinforce these observations and align with previous research. Even more, while uniquely focusing on younger patients with well-controlled skin lesions, a subgroup that has been largely overlooked in prior studies. They show that clinically relevant anxiety and depressive symptoms can still exist even in this population. It is still uncertain why these findings, along with the application of HADS, have not been systematically integrated into routine clinical care. We believe focusing on a well-defined population—young adults with moderate-to-severe psoriasis—can help increase clinician awareness and promote systematic psychological screening.

The bidirectional relationship between psoriasis and mental health is notable: psychological stress can trigger or worsen psoriatic flares, while the visible and chronic nature of the disease perpetuates emotional distress (19, 20). Psoriasis-related visible lesions and social stigma can amplify psychological distress, triggering social isolation and low self-worth, which can worsen disease course and severity, thereby perpetuating a detrimental feedback loop (19, 20). Thus, early identification and management of anxiety and depression symptoms are therefore crucial, not only to improve mental well-being but also to potentially mitigate disease severity and enhance treatment outcomes.

The identification of anxiety and depression can be challenging. The Hospital Anxiety and Depression Scale (HADS) is a widely validated instrument designed to assess psychological distress in clinical populations, with two separate subscales: HADS-A for anxiety and HADS-D for depression (10). Beyond dermatology, HADS has been applied in a variety of medical conditions, including cardiovascular disease, chronic inflammatory disorders, and cancer, where it often aligns well with formal psychiatric assessments, such as structured interviews and diagnostic questionnaires (21–23). In psoriasis specifically, studies comparing HADS results with psychiatric evaluation have shown moderate-to-strong concordance, supporting its utility as a rapid, reliable screening tool for identifying patients who may benefit from further mental health evaluation and intervention (6, 24).

With respect to treatment effects on psychological symptoms, the VOYAGE-2 study demonstrated that 24 weeks treatment with guselkumab significantly reduced anxiety and depression scores compared with both placebo and adalimumab (9). These improvements closely correlated with reductions in psoriasis severity and around 50% of improvement was attributed to guselkumab independently of PASI improvement (9). Similarly, the PHOENIX-2 study showed that 12 weeks treatment with ustekinumab led to a significant decrease in HADS scores in patients with moderate to severe psoriasis (25). While the psychological burden of psoriasis was recognized in these studies, the focus was on treatment efficacy, with psychological outcomes considered a secondary endpoint. The VOYAGE-2 and PHOENIX-2 studies included broader psoriasis populations with varying ages and comorbidities. Also, these studies focused on treatment efficacy; therefore, improvement in secondary psychological outcomes was somewhat expected, as highly effective drugs were used and both inflammation and skin burden were clearly and significantly reduced in a relatively short time (9, 25). In contrast, our study focused specifically on a younger cohort of psoriasis patients without comorbidities, all of whom were in stable phase of well-treated disease by a broad range of treatment modalities. In general, our results support and extend the findings of the VOYAGE-2 and PHOENIX-2 studies by demonstrating a discrepancy between dermatological treatment success and psychological well-being, particularly regarding anxiety and depression, emphasizing that even longstanding successful treatment does not eliminate this disease-related psychological burden.

To further extend our investigation, we aimed to exploratorily assess potential associations between psychological distress and inflammatory and metabolic markers. These markers were selected because systemic inflammation and metabolic dysregulation are recognized as key contributors to psoriasis comorbidities, and it is plausible that they may also play a role in the pathogenesis of psychological distress (26, 27). Evidence from other diseases further supports the potential link between these mechanisms and mental health outcomes (28, 29). Given the small size of our cohort, we adopted a careful, hypothesis-generating approach. Linear regression analyses did not reveal significant associations, which is not unexpected given the limited statistical power. However, random forest machine-learning models suggested that depressive symptoms (HADS-D) might be linked to inflammatory cytokines (IL-6, IL-23) and metabolic indicators (waist circumference, HOMA-IR, FIB-4), whereas anxiety symptoms (HADS-A) showed weaker and less consistent associations.

Psoriasis and depression share common inflammatory pathways, characterized by the overexpression of proinflammatory cytokines such as TNF, IL-1, IL-6, and IL-17 (30). In our previous work with this cohort, we demonstrated that certain cytokines and metabolic disturbances can persist despite clinically successful psoriasis treatment (13, 15), suggesting that systemic inflammation, metabolic dysregulation, and psychological distress often coexist and may be interconnected. In particular, IL-6 and IL-23 not only drive skin inflammation but also sustain systemic inflammatory activity (31). Metabolic dysregulation, especially related to visceral adiposity commonly observed in psoriasis, can amplify systemic inflammation, creating a self-perpetuating cycle (32). This chronic inflammatory state, together with metabolic abnormalities, may contribute to additional comorbidities, including cardiovascular disease, insulin resistance, and depression (32). Proinflammatory cytokines may also influence neurobiological pathways involved in mood regulation, providing a mechanistic link between psoriasis, metabolic health, and depressive symptoms (32–34). In contrast, anxiety might be more related to mechanisms beyond biological pathways. One important factor may be »psychological scarring«, the lasting emotional impact of living with a chronic, visible, and socially stigmatizing condition such as psoriasis. The chronic nature of the disease, combined with social misunderstanding or isolation, can leave a profound psychological imprint, increasing susceptibility to anxiety. Similar patterns have been observed in other chronic diseases, such as Crohn’s disease, where patients may develop complex post-traumatic stress disorder even during clinical remission (35).

Our study identified a tentative signal suggesting that residual systemic inflammation and metabolic dysregulation may contribute primarily to depressive symptoms, but not to anxiety. These findings should be interpreted cautiously, as they are hypothesis-generating and not yet confirmed. Larger studies are clearly needed to test these assumptions, and our results provide a rationale for further research into the role of residual inflammation and visceral adiposity in psychological distress among psoriasis patients.

This study has several limitations. Its cross-sectional design precludes causal inference and prevents assessment of changes in psychological distress over time. The small sample size and sex imbalance are important limitations. The absence of healthy controls and an active disease comparison group restrict evaluation of disease-related psychological impact, while recruitment from a single center in Slovenia limits national and international applicability. Although HADS is a validated tool, using more specific instruments such as PHQ4 and PHQ-9 (36) or GAD-7 could provide additional insight. Longitudinal interventional studies are needed to clarify the efficacy of therapeutic approaches, determine whether psychological distress predisposes to psoriasis relapse or vice versa, and explore biological, psychological, and social risk factors, particularly in younger patients. Finally, our observations regarding potential associations are hypothesis-generating and should be interpreted cautiously, serving primarily to guide future research.

In conclusion, our study highlights that even in patients with well-controlled psoriasis, a subset of patients continues to experience psychological distress. These results underscore the importance of integrating routine psychological assessment into the overall psoriasis care, suggesting that the younger, well-treated psoriasis patients should not be excluded or forgotten in this regard. Future studies should aim to elucidate the specific biological and psychosocial pathways underlying depression and anxiety in psoriasis and well-treated psoriasis, enabling more targeted and personalized interventions. Addressing the psychological burden alongside physical disease may ultimately improve overall outcomes and quality of life for this vulnerable population.

## Data Availability

The raw data supporting the conclusions of this article will be made available by the authors, without undue reservation.
